# Prevalence and Associated Risk Factors of Irritable Bowel Syndrome Among General Population: A Cross-Sectional Study in Qassim Region, Saudi Arabia

**DOI:** 10.7759/cureus.57493

**Published:** 2024-04-02

**Authors:** Ahmed S Almuzaini, Reema Almuzaini, Haifa N Alsaleem, Abdulsalam Alsuhaibani, Asma Alsohaibani, Raghad Alwehaibi, Lamees Alharbi, Ghada F Alotaiby, Ammar M ALAmmari

**Affiliations:** 1 College of Medicine, Qassim University, Buraydah, SAU; 2 College of Medicine, Unaizah College of Medicine and Medical Sciences, Qassim University, Buraydah, SAU; 3 Gastroenterology, King Fahad Specialist Hospital, Buraydah, SAU

**Keywords:** saudi arabia, rome iv questionnaire, painful bowel movement, ibs family history, irritable bowel syndrome, food allergy, abdominal pain

## Abstract

Background

Irritable bowel syndrome (IBS) affects 10-20% of the global population, primarily manifesting as functional issues leading to abdominal discomfort. Key contributors like genetics, psychological factors, weakened immunity, and environmental pollutants play significant roles. Regional variations exist, with prevalence rates ranging from 7-10% in certain areas like South Asia and the Middle East to as high as 20% in many Western countries.

Objective

The objective of this study is to assess the prevalence of irritable bowel syndrome (IBS) and its related risk factors among the general populace of the Qassim region, Saudi Arabia, aiming to offer valuable insights for healthcare planning and intervention strategies.

Methods

A cross-sectional descriptive study was conducted, utilizing a validated self-administered questionnaire among residents of the Qassim region aged over 18 years. The questionnaire included demographic information about the participants and the validated Rome IV questionnaire for IBS in adults. Ethical approval for the study was obtained from the Qassim Research Ethics Committee, and data analysis was conducted using R script language version 4.3.3. A significance level of p < 0.05 was employed to interpret the results.

Results

Overall, significant associations were observed between IBS diagnosis and food allergy (AOR = 2.34, 99% CI: 1.27-4.29), family history of IBS (Adjusted Odd Ratio (AOR) = 7.03, 99% CI: 3.51-15.74), and abdominal pain lasting more than six months (AOR = 2.54, 99% CI: 1.49-4.33).

Conclusion

This study highlights a high IBS prevalence (21.4%) in Saudi Arabia's Qassim region. While no overall soda-IBS link was found, males showed a protective effect. Significant associations were noted between food allergy, family history, and abdominal pain with IBS diagnosis, especially among females. Further research on gender disparities and familial and abdominal pain roles in IBS management is warranted.

## Introduction

Irritable bowel syndrome (IBS) affects 10-20% of the global population, posing a significant concern. It primarily manifests as functional rather than structural issues, leading to abdominal pain and discomfort. Key contributing factors include genetics, psychological elements like depression, weakened immunity, and environmental pollutants, notably air pollution [1]. The prevalence of IBS is estimated to be approximately 11.2% worldwide. However, there is significant regional variation in this figure. Some places, such as South Asia and the Middle East, report prevalence rates between 7% and 10%, while many Western countries record rates as high as 20% [2].

In a health promotion project in Iwaka, Japan, a study examined the occurrence and factors predicting IBS in the community. A total of 993 volunteers took part, with 61 (6.1%) diagnosed with IBS. The study linked alcohol consumption and depression as the primary factors associated with IBS [3]. IBS is recognized as one of the most prevalent chronic gastrointestinal disorders, distinguished by frequent abdominal pain and alterations in stool appearance or frequency [4]. Symptoms can vary greatly among individuals, with some experiencing severe symptoms and others only mild to moderate ones. Additionally, IBS often coexists with psychiatric conditions such as depression and anxiety [5].

For a considerable duration, IBS was believed to stem from psychological or emotional causes, attributed to the absence of structural abnormalities. However, a shift in understanding revealed its development to be influenced by both genetic predisposition and environmental factors. This shift in perspective was influenced by advancements such as understanding gut dysbiosis, especially small intestine bacterial overgrowth, which can lead to symptoms of IBS [6].

In a study aiming to explore the epidemiology of IBS among undergraduate students in Saudi Arabia, researchers conducted a cross-sectional study involving 767 undergraduates from February 2018 to June 2018. Data collection utilized self-administered online questionnaires. Findings revealed a prevalence of 15.8% among the study population, with poor lifestyle and dietary choices, prolonged stress, and depression identified as the primary causes [7].

Another cross-sectional study conducted in October 2020 targeted the adult Saudi population in the central region. Researchers distributed self-administered, semi-structured questionnaires online to facilitate data collection. With a sample size of 426 and a ± 5% margin of error and 95% confidence interval, the study found that 130 individuals met the Rome III criteria for IBS, indicating a prevalence of 30.5%. Despite the limited sample size, the study concluded that most IBS sufferers were female, experienced anxiety and depression, and exhibited reduced activities of daily living compared to the average person [8].

The primary pathophysiology behind the development of IBS remains incompletely understood. Several factors may contribute to its development, including visceral hypersensitivity, altered gut-brain connections, intestinal microbial alterations, mucosal inflammation, and genetic predisposition [9]. Previous studies on the prevalence of IBS in the Kingdom of Saudi Arabia, which ranged from 9% to 40%, mainly focused on school teachers, college students, and school-age children. These studies predominantly utilized earlier versions of the Rome criteria [9]. In many cases, IBS does not progress and does not lead to subsequent complications such as intestinal cancer, ulcers in the gastrointestinal tract mucous membrane, or inflammation.

In South Taiwan, a study was conducted involving 2,520 female university students to investigate the prevalence and associated risk factors of IBS. Using the Rome III criteria and a structured questionnaire for data collection, 1,894 students completed the questionnaire. Among them, 193 students were diagnosed with IBS, resulting in a prevalence rate of 10.1%. Those with IBS were observed to have lower quality of life in terms of diet and higher levels of stress compared to others [10].

Studies have revealed that the age group between 20 and 40 years old exhibits the highest frequency of IBS. Additionally, reports suggest that women are more prone to experiencing IBS compared to men. Genetic factors may also play a significant role in IBS, with up to 30% of cases being influenced by a patient's family history [11, 12]. IBS, characterized by functional rather than structural digestive tract issues, poses challenges due to the absence of anatomical changes. In May 2022, a University of Bangladesh study examined IBS prevalence among 300 randomly selected students using Rome III criteria. Shockingly, 39.3% were affected, with 77.3% lacking awareness of IBS, highlighting a significant awareness gap. Depression, poor nutrition, and prolonged anxiety correlated with higher prevalence [13]. In April 2022, a study on IBS prevalence and risk factors in two Egyptian medical faculties surveyed 182 students. Utilizing self-administered questionnaires and Rome III criteria, it found 27.5% with IBS. Many consumed excessive coffee and dairy but lacked fruits and vegetables. Depression, anxiety, and poor diet were linked to IBS [14].

A cross-sectional study at KRL Hospital, Islamabad, from March to May 2022, revealed 48.66% experiencing defecation-related abdominal pain. High tea (54%), coffee (27%), and soft drink (19%) consumption were noted. Timely screening and medical intervention were crucial [15]. A cohort study investigated the link between Non-Alcoholic Fatty Liver Disease (NAFLD) and IBS. Among 396,838 participants, NAFLD patients had a 13% higher IBS risk over a 12.4-year median follow-up, confirming the association [16]. In the Middle East, a cross-sectional study among North Jordanian medical students found a 30.9% IBS prevalence. Factors such as elevated anxiety scores, poor sleep patterns, female gender, and living in school dorms were associated with IBS [17].

Moreover, IBS can exacerbate already exorbitant medical expenses and diminish work productivity and health-related quality of life (QOL). Studies have demonstrated that patients with IBS typically experience a significantly lower quality of life [18]. Research conducted in Saudi Arabia indicates that 28% of referrals to gastroenterologists and 12% of primary healthcare visits are linked to IBS. Alarmingly, only 15% of these patients seek medical care [18]. Mendelian randomization was employed to investigate the association between alcohol consumption, cigarette smoking, and common gastrointestinal conditions, using data from the UK Biobank and the FinnGen study. The study found a link between IBS and both alcohol consumption and cigarette smoking. Notably, cigarette smoking was associated with 20 out of 24 gastrointestinal conditions studied, significantly more than alcohol consumption [19]. A study conducted in Al-Qunfudah Governorate, Saudi Arabia in 2023 aimed to estimate the prevalence of IBS and identify associated risk factors. Among 335 adults sampled from the general population, the prevalence of IBS was 30.4%, with females exhibiting a higher prevalence at 55.9% compared to males at 44.1%. The analysis highlighted prominent risk factors such as being single, having a family history of IBS, and experiencing high levels of anxiety [20].

Numerous studies have explored the occurrence and risk elements associated with IBS among educators, university students, and secondary school students in the Qassim region. Nonetheless, as far as our knowledge extends, no research has exclusively focused on the broader populace of the Qassim region. Hence, the objective of this study is to assess the prevalence of IBS and its related risk factors among the general populace of the Qassim region, Saudi Arabia, aiming to offer valuable insights for healthcare planning and intervention strategies.

## Materials and methods

A cross-sectional descriptive study was conducted in the Qassim region, Saudi Arabia. The sample size was calculated using the Raosoft sample size calculator [21], considering the estimated population of the Qassim region as 1,016,765 according to reports from the Ministry of Interior. It was determined that 385 participants were required for this investigation to achieve a 95% confidence interval, a 5% margin of error, and a predetermined prevalence of 50%.

A validated self-administered questionnaire distributed via Google Forms was utilized. The study achieved a sample size of 434 participants, comprising both males and females aged over 18 years residing in the Qassim region, who voluntarily consented to participate. However, three individuals were excluded from the study due to non-Saudi nationality, thus rendering the figure incomparable, and 29 were excluded because they were non-residents of the Qassim region. This resulted in a reduction of the sample size to 402 participants.

The questionnaire comprised two sections: the first section gathered demographic information about the participants, while the second section included the validated Rome IV questionnaire for IBS in adults. The questionnaire was presented in Arabic. Participants received a clear explanation of the study's purpose, and the questionnaire did not request any personal information. A pilot study was undertaken to validate the questionnaire. Random individuals from the community were selected, and the questionnaire was tested. Based on the feedback received, questions were refined for clarity and comprehension. The Rome IV diagnostic questionnaire, a previously published and validated tool by the Rome Foundation, constituted the second part of the survey.

Ethical approval for the study was obtained from the Qassim Research Ethics Committee (reference number 607/45/10229), demonstrating a commitment to upholding the highest ethical standards. Data collection strictly adhered to these guidelines, ensuring anonymity and protecting participants' privacy through informed consent procedures. Data analysis was conducted using R, a script language version 4.3.3.

## Results

Descriptive statistics

Descriptive statistics and inferential statistics are presented in Table 1. The data were segregated into those diagnosed with IBS and those not diagnosed with IBS. The data are presented in the form of frequencies, percentages, and p-values, which were obtained through chi-test and cross-tabulation. Please refer to Table 1 for further statistics. Overall, significant associations were observed regarding gender, age, weight, food allergy, history of IBS, chronic illness, abdominal pain, painful bowel movement, stool consistency, stool frequency, and experiencing pain for more than six months. These variables were later subjected to logistic regression analysis.

**Table 1:** Overall descriptive statistics IBS: Irritable bowel syndrome

Variable	Characteristics	No diagnosis of IBS	Diagnosed with IBS	p-value
Nationality	Saudi	316 (78.6%)	86 (21.4%)	
Gender	Female	248 (61.7%)	80 (19.9%)	0.002
	Male	68 (16.9%)	6 (1.5%)	
Age	18-29 years	150 (37.3%)	32 (8%)	0.049
	30-39 years	57 (14.2%)	11 (2.7%)	
	40-49 years	81 (20.1%)	29 (7.2%)	
	Over 50 years	28 (7%)	14 (3.5%)	
Marital status	Single	148 (36.8%)	35 (8.7%)	0.086
	Married	152 (37.8%)	51 (12.7%)	
	Divorced	6 (1.5%)	0 (0%)	
	Widow	10 (2.5%)	0 (0%)	
Education	Secondary or less	39 (9.7%)	9 (2.2%)	0.884
	Diploma	30 (7.5%)	10 (2.5%)	
	Bachelor	233 (58%)	64 (15.9%)	
	Postgraduate	14 (3.5%)	3 (0.7%)	
Occupation	Unemployed	65 (16.2%)	19 (4.7%)	0.819
	Student	92 (22.9%)	21 (5.2%)	
	Employee	145 (36.1%)	41 (10.2%)	
	Retired	14 (3.5%)	5 (1.2%)	
Parents Status	Dead (one or both)	120 (29.9%)	35 (8.7%)	0.282
	Divorced	6 (1.5%)	4 (1%)	
	Living together	190 (47.3%)	47 (11.7%)	
Family Income	< 5000 SR	39 (9%)	9 (2.2%)	0.759
	5000-10000 SR	97 (24.1%)	30 (7.5%)	
	> 10000 SR	183 (45.5%)	47 (11.7%)	
Family members	Less than 5	75 (18.7%)	21 (5.2%)	0.851
	5-10 members	217 (54%)	60 (14.9%)	
	> 10 members	24 (6%)	5 (1.2%)	
Weight	Underweight	18 (4.5%)	11 (2.7%)	0.049
	Right weight	178 (44.3%)	40 (10%)	
	Overweight	120 (29.9%)	35 (8.7%)	
Physically active	0 days	158 (39.3%)	37 (9.2%)	0.24
	1-3 days	123 (30.6%)	42 (10.4%)	
	4-7 days	35 (8.7%)	7 (1.7%)	
Sleep	Less than 5 hours	20 (5%)	6 (1.5%)	0.527
	5-8 hours	252 (62.7%)	64 (15.9%)	
	> 9 hours	44 (10.9%)	16 (4%)	
Eatery	Home	273 (67.9%)	74 (18.4%)	0.984
	Restaurants	40 (10%)	11 (2.7%)	
	Others	3 (0.7%)	1 (0.2%)	
Meals per day	One meal	54 (13.4%)	13 (3.2%)	0.625
	2-3 meals	244 (60.7%)	70 (17.4%)	
	> 3 meals	18 (4.5%)	3 (0.7%)	
Eat breakfast	0 days	59 (14.7%)	15 (3.7%)	0.203
	1-3 days	128 (31.8%)	27 (6.7%)	
	4-7 days	129 (32.1%)	44 (10.9%)	
Eat fruits	Did not eat fruits	102 (25.4%)	28 (7%)	0.735
	1-3 times	163 (40.5%)	47 (11.7%)	
	> 3 times	51 (12.7%)	11 (2.7%)	
Eat vegetables	Did not eat vegetables	38 (9.5%)	11 (2.7%)	0.977
	1-3 times	180 (44.8%)	49 (12.2%)	
	> 3 times	98 (24.4%)	26 (6.5%)	
Drink soda	Did not drink soda	142 (35.3%)	49 (12.2%)	0.064
	1-3 times	122 (30.3%)	30 (7.5%)	
	> 3 times	52 (12.9%)	7 (1.7%)	
Mental health	Never	23 (5.7%)	4 (1%)	0.448
	Rarely	76 (18.9%)	16 (4%)	
	Sometimes	157 (39.1%)	45 (11.2%)	
	Most of the time	60 (14.9%)	21 (5.2%)	
Food allergy	No	227 (56.5%)	42 (10.4%)	< 0.001
	Yes	48 (11.9%)	28 (7%)	
	I do not know	41 (10.2%)	16 (4%)	
Spicy foods	No	154 (38.3%)	48 (11.9%)	0.244
	Yes	162 (40.3%)	38 (9.5%)	
IBS History	No	94 (23.4%)	6 (1.5%)	<0.001
	Yes	164 (40.8%)	77 (19.2%)	
	I do not know	58 (14.4%)	3 (0.7%)	
Chronic Illness	No	277 (68.9%)	67 (16.7%)	0.023
	Yes	39 (9.7%)	19 (4.7%)	
Abdominal pain	Every day	0 (0%)	4 (1%)	< 0.001
	Less than one day a month	100 (24.9%)	13 (3.2%)	
	Most day	27 (6.7%)	18 (4.5%)	
	Multiple times per day	6 (1.5%)	4 (1%)	
	Once a week	25 (6.2%)	8 (2%)	
	One day a month	46 (11.4%)	13 (3.2%)	
	Two to three days a month	77 (19.2%)	13 (3.2%)	
	Two to three days a week	35 (8.7%)	13 (3%)	
Painful bowel movement	0% never	97 (24.1%)	7 (1.7%)	< 0.001
	10%	74 (18.4%)	18 (4.5%)	
	20%	25 (6.2%)	11 (2.7%)	
	30%	32 (8%)	8 (2%)	
	40%	16 (4%)	6 (1.5%)	
	50%	33 (8.2%)	9 (2.2%)	
	60%	17 (4.2%)	3 (0.7%)	
	70%	9 (2.2%)	7 (1.7%)	
	80%	3 (0.7%)	9 (2.2%)	
	90%	2 (0.5%)	1 (0.2%)	
	100% always	8 (2%)	7 (1.7%)	
Soft or Hard stool	0% never	117 (29.1%)	13 (3.2%)	< 0.001
	10%	65 (16.2%)	16 (4%)	
	20%	25 (6.2%)	7 (1.7%)	
	30%	27 (6.7%)	8 (2%)	
	40%	17 (4.2%)	4 (1%)	
	50%	21 (5.2%)	13 (3.2%)	
	60%	14 (3.5%)	3 (0.7%)	
	70%	14 (3.5%)	6 (1.5%)	
	80%	6 (1.5%)	8 (2%)	
	90%	3 (0.7%)	3 (0.7%)	
	100% always	7 (1.7%)	5 (1.2%)	
Frequent or less stool	0% never	118 (29.4%)	15 (3.7%)	0.001
	10%	65 (16.2%)	18 (4.5%)	
	20%	33 (8.2%)	15 (3.7%)	
	30%	26 (6.5%)	4 (1%)	
	40%	24 (6%)	3 (0.7%)	
	50%	19 (4.7%)	13 (3.2%)	
	60%	10 (2.5%)	6 (1.5%)	
	70%	7 (1.7%)	1 (0.2%)	
	80%	7 (1.7%)	5 (1.2%)	
	90%	3 (0.7%)	2 (0.5%)	
	100% always	4 (1%)	4 (1%)	
Pain more than 6 months	No	226 (56.2%)	38 (9.5%)	<0.001
	Yes	90 (22.4%)	48 (11.9%)	
Drink Soda (Re-coded)	No	142 (35.3%)	49 (12.2%)	0.047
	Yes	174 (43.3%)	37 (9.2%)	
Food allergy (Re-coded)	No	268 (66.7%)	58 (14.4%)	< 0.001
	Yes	48 (11.9%)	28 (7%)	
IBS History (Re-coded)	No	152 (37.8%)	9 (2.2%)	<0.001
	Yes	164 (40.8%)	77 (19.2%)	
Painful bowel movement (Re-coded)	No	107 (26.6%)	15 (3.7%)	0.003
	Yes	209 (52%)	71 (17.7%)	
Soft or Hard stool (Re-coded)	No	127 (31.6%)	21 (5.2%)	0.007
	Yes	189 (47%)	65 (16.2%)	
Frequent or less stool (Re-coded)	No	125 (31.1%)	21 (5.2%)	0.01
	Yes	191 (47.5%)	65 (16.2%)	

Several variables were recoded into binary variables to enable logistic regression, with only significant variables depicted in Table 1. Among those diagnosed with IBS, 9.2% had drunk soda one week prior to data collection, 7% had an intolerance or allergy to a certain type of food, 19.2% had a family history of IBS, 17.7% had experienced painful bowel movements, and 16.2% had either experienced soft or hard stools, or frequent or less stools.

Multivariate analysis

Significant associations were reported between drinking soda, food allergy, history of IBS, painful bowel movement, and experiencing pain for more than six months with IBS, as shown in Table 2.

**Table 2:** Association of significant independent variables with the diagnosis of IBS Notes: *p<0.1; **p<0.05; ***p<0.01. The figure outside the brackets shows the AOR, while the figures inside the brackets show the confidence intervals. Confidence level: 95%

	Dependent variable: Diagnosed with IBS		
Independent variables	Overall model (1)	Male model (2)	Female model (3)
(1) Drinking Soda	0.78 (0.45, 1.34)	0.06** (0.002, 0.65)	1.02 (0.58, 1.80)
(2) Food allergy	2.34*** (1.27, 4.29)	9.92* (0.84, 265.89)	2.20** (1.15, 4.17)
(3) History of IBS	7.03*** (3.51, 15.74)	20.34** (2.00, 780.60)	6.04*** (2.87, 14.30)
(4) Pain more than six months	2.54*** (1.49, 4.33)	4.05 (0.49, 46.91)	2.55*** (1.46, 4.47)
Constant	0.04*** (0.02, 0.09)	0.018*** (0.0005, 0.16)	0.046*** (0.02, 0.10)
Observations	402	74	328
Log Likelihood	-173.9	-12.66	-156.34
Akaike Inf. Crit.	357.8	35.32	323.68

## Discussion

In this investigation, the prevalence of IBS in the Qassim region of the Kingdom of Saudi Arabia was found to be 21.4%. This rate contrasts with the 30.5% documented among undergraduate students in Saudi Arabia by Aljammaz et al. [8] and the 15.8% among the adult population in the central region reported by AlButaysh et al. [7].

Regarding the association between drinking soda and IBS diagnosis, the AOR for the overall model suggests no significant link. This implies that, on the whole, soda intake does not serve as a significant predictor of IBS diagnosis. This observation contradicts prior studies [7, 10, 12-14] linking poor dietary habits, including frequent soda consumption, with IBS. However, when examining gender disparities, the AOR for males was significant at a 95% confidence level. Male respondents who consumed soda were approximately 0.06 times less likely to receive an IBS diagnosis compared to their non-consumer counterparts, suggesting a protective effect of soda consumption against IBS diagnosis among males. Conversely, the AOR for females was non-significant, indicating no substantial association between soda consumption and the likelihood of IBS diagnosis among females in our study. Overall, these findings underscore a potential gender discrepancy in the relationship between soda consumption and IBS diagnosis, with a significant effect observed in males but not females.

In this investigation, the AOR for the overall model reveals a notable association between food allergy and the likelihood of IBS diagnosis. Individuals experiencing food allergy had approximately 2.34 times higher odds of receiving an IBS diagnosis compared to those without such allergies, with a confidence level of 99%. However, when analysing gender disparities, the AOR for males was non-significant, indicating no significant association between food allergy and IBS diagnosis among males. Conversely, for females, the AOR remained significant at a 99% confidence level. Females with food allergies were approximately 2.20 times more likely to be diagnosed with IBS compared to their non-allergic counterparts. This suggests a strong association between food allergy and IBS diagnosis among females, which was not observed among males in our study. These findings align with prior research [8, 11, 16, 19] all reporting a higher prevalence of IBS among females compared to males.

A substantial association was found between a family history of IBS and IBS diagnosis. Individuals with such a family history had approximately 7.03 times higher odds of receiving an IBS diagnosis compared to those without, with a confidence level of 99%. Upon exploring gender disparities, the AOR for males exhibited a notably higher magnitude, indicating a stronger association between a family history of IBS and the likelihood of an IBS diagnosis among males. Specifically, the odds of being diagnosed with IBS among males with a family history were approximately 20.34 times higher compared to those without, with a confidence level of 95%. Similarly, for females, the AOR remained significant, albeit slightly lower than that for males. Females with a family history of IBS were approximately 6.04 times more likely to receive an IBS diagnosis compared to those without, with a confidence level of 99%. These findings align with prior research [11, 19], which associated family history with IBS, particularly among females. Overall, our results indicate that having a family history of IBS serves as a robust predictor of an IBS diagnosis, with males exhibiting a notably heightened risk compared to females.

Experiencing abdominal pain was significantly associated with IBS diagnosis, confirming previous findings. This study corroborates previous research by [4, 14], suggesting a potential link between frequent abdominal pain and IBS. Individuals reporting abdominal pain had approximately 2.54 times higher odds of receiving an IBS diagnosis compared to those without such symptoms, with a confidence level of 99%. Upon scrutinising gender disparities, the AOR for males was non-significant, indicating no substantial association between abdominal pain and the likelihood of IBS diagnosis among males. However, for females, the AOR remained significant at a 99% confidence level. Female individuals experiencing abdominal pain were approximately 2.55 times more likely to be diagnosed with IBS compared to females without abdominal pain. This suggests a robust association between abdominal pain and the likelihood of IBS diagnosis among females, which was not observed among males in our study.

This study has encountered several limitations that warrant caution when applying the findings to the wider population of the Qassim region, Saudi Arabia. Firstly, there was an uneven distribution of genders, with approximately 82% of the dataset consisting of females and the remaining being males. As a result, it is not advisable to generalize the findings solely based on gender. Furthermore, the original dataset included individuals who were not residents of the Qassim region. In subsequent studies, it would be prudent to include this category of non-residents for comparison, especially concerning issues like IBS and food allergies. This addition would allow for comparisons across different culinary traditions from various regions within the Kingdom of Saudi Arabia, thereby enriching the study's scope. Additionally, future research should classify different types of food allergies, as individuals may react differently to various food categories.

Based on our findings, there's a notable prevalence of IBS in the Qassim region of Saudi Arabia, at 21.4%. Ongoing research is crucial to understand IBS epidemiology and risk factors, especially regionally and demographically. The gender disparity in the link between soda consumption and IBS warrants further exploration. Investigating factors contributing to this gender gap and its impact on IBS risk is essential. The 2.34-fold increase in IBS diagnosis among those with food allergies underscores the need for allergy screening in IBS management. While females with food allergies have a higher likelihood of IBS, it's not observed in males, indicating gender-specific complexities in IBS. Recognising family history's impact on IBS risk is vital in patient assessments, potentially improving outcomes. Longitudinal studies exploring familial predispositions, lifestyle factors, and gastrointestinal symptoms may offer insights into IBS pathophysiology. Abdominal pain is a key indicator of gastrointestinal issues, particularly in females. Thorough assessment and management are crucial for early intervention in those at risk of IBS.

## Conclusions

This study in the Qassim region of Saudi Arabia revealed a high IBS prevalence of 21.4%. While no significant link was found between soda consumption and IBS overall, a gender difference showed a protective effect in males. Notably, food allergies showed a significant correlation with IBS, especially in females. Family history of IBS was a strong predictor, with males at higher risk. Additionally, a significant association was found between abdominal pain and IBS, particularly in females, emphasizing the need for further research on gender disparities, familial influences, and abdominal pain in effective IBS management.
